# A 19-year-old male with palpitations

**DOI:** 10.4103/0974-2700.41792

**Published:** 2008

**Authors:** Shailendra Upadhyay, Shweta Upadhyay

**Affiliations:** 1 Department of Pediatric Cardiology, Long Island Jewish Medical Center, Schneider Children's Hospital, 269-01, 76^th^ Avenue, New Hyde Park, NY 11040, USA; 2 Department of Medicine, Winthrop University Hospital, 222 Station Plaza, Mineola, NY 11040, USA

**Keywords:** Arrhythmogenic right ventricular dysplasia, epsilon waves, palpitations

## Abstract

A 19-year-old male presented to the emergency department (ED) following intermittent episodes of palpitations. Classical “epsilon waves” noted on his initial electrocardiogram prompted an evaluation for arrhythmogenic right ventricular dysplasia (ARVD). The diagnosis was confirmed with magnetic resonance imaging of the heart and stress test. A prompt recognition and management of this condition in the ED helped prevent significant mortality that may be associated with ARVD.

## CASE HISTORY

A 19-year-old male presented to the emergency department (ED) following 30 min of intermittent palpitations while watching television at home. While being seen and evaluated in the ED, his palpitations had resolved. He was a robust adolescent with a heart rate of 68 bpm and blood pressure of 100/60 mm Hg. Rest of his physical examination and cardiac examination revealed no abnormality.

His past medical history was unremarkable. His family history was unremarkable except that one of his uncle's from father's side has had a pacemaker/implantable cardioverter defibrillator (ICD) implanted for episodes of ventricular tachycardia. His uncle was 34 years old at the time of ICD implant and lives in Italy. Detailed information regarding the indications for ICD implant could not be obtained. He denied using illicit drugs of abuse, over the counter medications or excessive caffeine ingestion. A urine toxicology screen and serum electrolytes (sodium, potassium, magnesium, and calcium) were normal.

**Figure 1:**
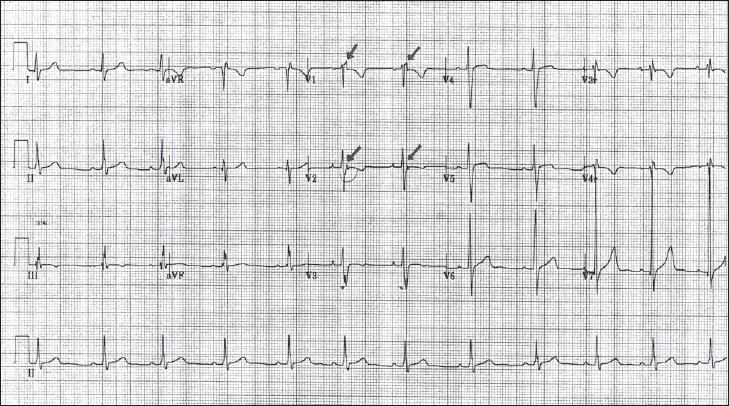
ECG on presentation in the emergency department (ED) showing a normal sinus rhythm at 68 bpm, incomplete right bundle branch block (RBBB), “T” wave inversions from V1 to V3, and epsilon waves (arrows) noted in leads V1 and V2

His electrocardiogram (ECG) revealed [Figure 1]: A normal sinus rhythm at 68 bpm, incomplete right bundle branch block (RBBB).

The findings were thought to be within normal limits for age. The patient was observed for few hours in the ED and then discharged home with instructions to follow up with the cardiologist.

Within a week he was evaluated by cardiology when the ECG was noted to have “epsilon waves” in leads V1 and V2 with T wave inversions in leads V1 to V3. An evaluation for arrhythmogenic right ventricular dysplasia (ARVD) was performed. A two-dimensional ECG with Doppler was normal. Magnetic resonance imaging (MRI) of heart revealed focal thinning at the right ventricular outflow tract. An exercise stress test demonstrated two episodes of four beat runs of ventricular tachycardia with left bundle branch block (LBBB) morphology and right axis deviation. He was diagnosed with ARVD based on clinical criteria and started on Atenolol. He had no further recurrence of his symptoms at 6 months of follow-up.

## DISCUSSION

Arrhythmogenic right ventricular dysplasia may be a lethal cause of cardiac disease that was first described by Fontaine * et al*.[1,2] It is a disorder where normal myocardium in the right ventricular free wall or the right ventricular outflow tract is replaced by fibrofatty tissue. Incidence of ARVD may range from 6 to 44 per 10,000 persons in different populations. ARVD is responsible for 3-4% sports deaths and 5% sudden deaths in patients under 65 years of age.[3,4]

Nine genetic loci associated with this disease and mutations in genes at 3 loci have been identified. Mutations in genes encoding desmoplakin and plakoglobin suggest that altered integrity at cardiac myocyte cell-cell junctions possibly lead to myocyte degeneration and death. Repair consists of replacement of myocardium by adipose and fibrous tissue. Gene mutations encoding the cardiac ryanodine receptor suggest that cytoplasmic calcium overloading may be responsible for arrhythmias characteristic of ARVD.[5]

Given its common occurrence and first presentation often being in ED, it is important for ED physicians to be aware of its presentation, classical ECG findings, and management. Various major and minor criteria have been devised for diagnosis of ARVD[6–8] [Table 1]. There are characteristic ECG findings present in 50-90% of patients with ARVD. These findings include T wave inversions in precordial leads, epsilon waves, ventricular tachycardia with LBBB morphology and occasionally polymorphic or RBBB morphology.[9–12] Physical examination may range from being normal in majority of patients, to fatigue, syncope, and even sudden cardiac death.

**Table 1:** Adapted from task force of the working group for myocardial and pericardial disease of the European Society of Cardiology and of the scientific council on cardiomyopathies of the International Society and Federation of Cardiology

Criteria	Global or regional dysfunction and structural alterations	Repolarization of abnormalities	Depolarization or conduction abnormalities	Arrhythmias	Family history
Major	Sever dilatation and reduction in the RV ejection fraction with no or only mild left ventricular impairment or Localized RV aneurysms (akinetic-dyskinetic areas of diastolic bulding)	None	Epsilon waves* or localized prolongation (110 ms) of the QRs complex in precordial leads (V_1_, V_2_ or V_3_)	Sustained left bundle-branch-block type of VT (as determined with electrocardiography, Holter monitoring, or exercise testing)	Familial disease confirmed at necropsy or surgery
Minor	Minor global RV dilation or ejection fraction reduction with normal LV, or Mild segmental dilation of the RV, or Regional RV hypokinesia	Inverted T waves in the right precordial leads beyond V_1_ (patient >12 years, in the absence of a RBBB)	Late potentials (signal-averaged electrocardiography)	Frequent ventricular extrasystoles with left bundle-branch-block morphology (>1000 per 24h, as seen with Holter monitoring)	Family history of premature sudden death (<35 years) caused by suspected RVD or Family history (clinical diagnosis based on current criteria)

* FOR DIAGNOSIS OF ARVD, THE PATIENT MUST HAVE TWO MAJOR CRITERIA, ONE MAJOR AND TWO MINOR CRITERIA, OR FOUR MINOR CRITERIA, OUR PATIENT HAD ONE MAJOR (EPSILON WAVES), AND TWO MINOR (INVERTED T WAVES V1-V3 AND POSITIVE FAMILY HISTORY OF ICD IMPLANT). HE ALSO HAD TYPICAL VENTRICULAR TACHYCARDIA MORPHOLOGY DURING THE STRESS TEST-MARGINALLY MEETING THE MAJOR-ARRHYTHIMIA CRITERIA. IN ADDITION OUR PATIENT HAD FOCAL THINNING NOTED AT THE RIGHT VENTRICULAR OUTFLOW TRACT-ON MRI, THIS CAN POSSIBLY ALSO BE INCLUDED AS ANOTHER MINOR CRITERION. RV = RIGHT VENTRICLE, LV = LEFT VENTRICLE

Postexcitation of myocytes in the right ventricle causes “epsilon waves” on ECG. They appear as small deflections just beyond the QRS complex. Young patients with ventricular tachycardia or syncope and epsilon waves on the ECG may have ARVD. In ARVD fat replaces myocytes, producing areas of the viable myocytes surrounded by fat. This leads to delay in excitation of some of the myocytes of the right ventricle that are noted as small deflections during the ST segment of the ECG.[13]

Epsilon waves may also be seen with posterior myocardial infarction, right ventricular infarction, and sickle cell disease with right ventricular hypertrophy due to pulmonary arterial hypertension.[1,14]

Echocardiogram with or without contrast may help delineate certain features of ARVD such as ventricular aneurysms and areas of dyskinesis in the triangle of dysplasia; however, most often these studies are normal.[15] Exercise stress testing may provoke the ventricular tachycardia with typical morphology (such as in our patient) on rare occasions, but often the results are normal. Heart MRI may be able to provide noninvasive localization of structural changes.[16] Diagnostic criteria for ARVD are clinical and endomyocardial biopsy (EMB) is generally not indicated. It may some times be required to distinguish it from other forms of myocarditis. Due to the focal nature of myocardial involvement often the results of EMB may not be useful.

Our patient was diagnosed with ARVD based on presence of epsilon waves on his ECG, T wave inversions from leads V1 to V3, positive family history of ICD implantation, brief runs of ventricular tachycardia with LBBB morphology and right axis deviation on his stress test, and evidence of myocardial thinning noted at right ventricular outflow tract on MRI of his heart. Diagnostic criteria of ARVD are given in Table 1.[17] He was started on oral Atenolol per the recommendations of current treatment options for ARVD. Beta-blockers possibly work in suppressing the ventricular tachycardia by virtue of their catecholamine blocking properties.[18,19] Our patient had no further recurrence of his tachyarrhythmia at 6 months of follow-up. The natural history of ARVD is associated with an annual mortality of 2.3%. Many patients may develop congestive heart failure; however, the most common modality of death is from ventricular tachycardia.[20] A careful periodic follow-up is required for development of symptoms of congestive heart failure or arrhythmia.

It is important to recognize these subtle but important findings on initial ECG. ARVD may have grave outcomes if the findings are not quickly identified and acted upon.
